# Ruxolitinib for the treatment of SARS-CoV-2 induced acute respiratory distress syndrome (ARDS)

**DOI:** 10.1038/s41375-020-0907-9

**Published:** 2020-06-17

**Authors:** Andreas Neubauer, Thomas Wiesmann, Claus F. Vogelmeier, Elisabeth Mack, Chrysanthi Skevaki, Christine Gaik, Christian Keller, Jens Figiel, Kristina Sohlbach, Caroline Rolfes, Harald Renz, Hinnerk Wulf, Andreas Burchert

**Affiliations:** 1 Department of Medicine, Hematology, Oncology, Immunology, University Hospital Giessen and Marburg, and Philipps University Marburg, Baldinger Strasse 1, Marburg, Germany; 2 Department of Anesthesiology and Intensive Care Medicine, University Hospital Giessen and Marburg, and Philipps University Marburg, Baldinger Strasse 1, Marburg, Germany; 3 Department of Medicine, Pulmonary and Critical Care Medicine, University Hospital Giessen and Marburg and Philipps University Marburg, Member of the German Center for Lung Research (DZL), Baldinger Strasse 1, Marburg, Germany; 4 Institute of Laboratory Medicine, Universities of Giessen and Marburg Lung Center (UGMLC), Philipps University Marburg, German Center for Lung Research (DZL), Marburg, Germany; 5 Institute of Virology, University Hospital Giessen and Marburg, and Philipps University Marburg, Hans-Meerwein-Strasse 2, Marburg, Germany; 6 Department of Diagnostic and Interventional Radiology, University Hospital Giessen and Marburg, and Philipps University Marburg, Baldinger Strasse 1, Marburg, Germany; 7 Department of Anesthesiology, Critical Care Medicine, Emergency Medicine and Pain Therapy, Klinikum Kassel, Mönchebergstraße 41-43, Kassel, Germany

**Keywords:** Translational research, Innate immunity

Since its outbreak in December 2019 in Wuhan, China [1], the novel coronavirus, SARS-CoV-2, has created a dramatic global health and economic crisis. COVID-19 is the disease caused by SARS-CoV-2. In most cases, COVID-19 is associated with mild respiratory symptoms. However, in ~15% of the patients, hospitalization is required, and about 5% of patients develop severe lung injury that can result in acute respiratory distress syndrome (ARDS). ARDS may be accompanied by sepsis and septic shock, and multiorgan failure, including acute kidney injury and cardiac injury. Older age, obesity, pulmonary and other comorbidities are risk factors for higher mortality [2, 3]. It has also been reported that the extent of inflammation—mirrored by peripheral blood cytokine levels—is associated with a worse outcome [4].

LaRosée et al. describe a very interesting series of 14 COVID-19 patients with heavy inflammatory syndrome treated successfully with ruxolitinib [5]. As COVID-19 induces significant burden to societies worldwide, appropriate treatment also for COVID-19 associated ARDS would be beneficial. However, in the 14 patients described in this series, only one patient was mechanically ventilated before start of ruxolitinib.

We were interested whether inhibition of Janus kinases could also revert overt ARDS in COVID-19.

**Fig. 1:**
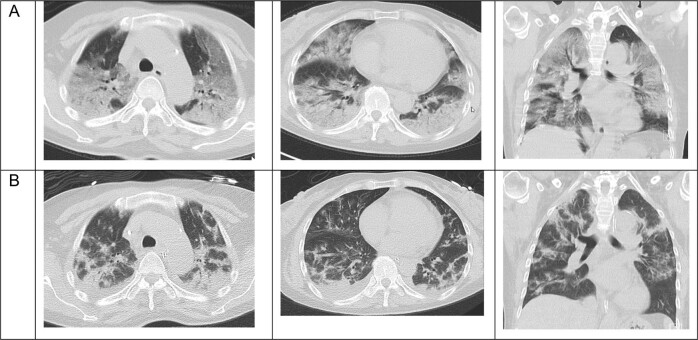
Ruxolitinib clears lung infiltration in SARS-CoV-2 induced ARDS. **a** Chest CT scans of the patient before transfer to the ICU reveals diffuse bilateral ground glass opacities, consolidations, and motion artifacts due to tachypnea. **b** After 11 days of treatment with ruxolitinib, markedly decreased ground glass opacities and motion artifacts, less pronounced regression of consolidations, and atelectasis.

**Fig. 2:**
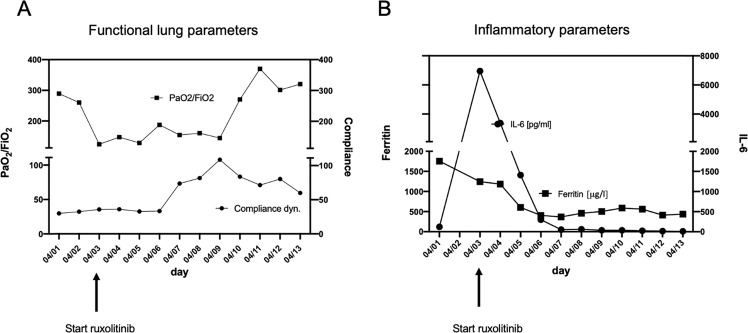
Ruxolitinib improves functional and inflammatory parameters in SARS-CoV-2 induced ARDS. Functional lung parameters (**a**) and laboratory parameters (**b**; ferritin and IL6) during treatment with ruxolitinib in a case of COVID-19 related ARDS.

We report on our first case of a 65-year old Asian woman with COVID-19-induced ARDS that was successfully treated with ruxolitinib. The patient presented with progressive dyspnea (respiratory rate >40/min, SpO2 of 60% on ambient air) and a history of fever for 3 days. Although there was no pre-existing disease in this patient, her respiratory distress deteriorated rapidly and she had to be intubated 3 hours after first contact in the emergency room. Computed tomography of the chest revealed extensive bilateral ground glass opacities and consolidations (Fig. 1). The patient was transferred to the ICU and intubated immediately. SARS-CoV-2 infection was confirmed by combined E- and S-specific PCR (RealStar^®^ SARS-CoV-2 RT-PCR Kit, Altona Diagnostics, Hamburg, Germany) from a nasopharyngeal swab. The initial laboratory investigations revealed no abnormalities except for a lymphopenia of 5,4% (reference value 20–44%), and elevated levels for LDH (928 U/l, reference value < 247 U/l), ferritin (1756 μg/l, reference value 15–400 μg/l) and IL-6 (120 pg/ml, reference value <7 pg/ml) and a high sensitive troponin I (39,8 ng/l, reference value <15,8 ng/l), parameters suggesting an adverse clinical course [4]. Twelve hours later, the respiratory status deteriorated to a Horowitz index (paO2/FiO2) of 110 under pressure controlled ventilation (PCV) with a positive end expiratory pressure (PEEP) of 16mBar, a plateau pressure (P Peak) of 31mBar and a set respiratory rate of 30/min. To achieve an adequate perfusion pressure, norepinephrine was commenced at 4–16 µg/min. Prone positioning was performed on the sedated patient for intervals of 24 h. As a bacterial superinfection was suspected due to worsening of gas exchange, leukocytosis and substantial increase of CRP and procalcitonin, antibiotic treatment with meropenem was started (with dose adjustments based on therapeutic drug monitoring). Within a few hours NT-proBNP and TNI values increased substantially and a severe left ventricular dysfunction was diagnosed. The overall prognosis of this patient was considered to be very poor [6]. After counseling the ethics committee at our institution, an experimental treatment with 10 mg ruxolitinib BID was started. The drug was administered via a nasogastral tube. In parallel, standard of care treatment was continued. During the following days, the Horowitz index improved continuously under assisted spontaneous ventilation with a PEEP of 12mBar and P_ASB_ of 9mBar with 24 h intervals of prone and supine positioning facilitated by sedation with dexmedetomidine, propofol and sufentanil (Fig. 2a). At day 8 after ICU admission, percutaneous dilatational tracheotomy was performed and the patient was intermittently weaned from the respirator starting at day 10. RT-PCR from serum samples before ruxolitinib (Ct = 35.84) and 5 days after start of ruxolitinib (Ct = 35.86) suggested that SARS-CoV-2 viremia did not increase. IL6 and ferritin levels returned to normal (Fig. 2b).

To the best of our knowledge, we here describe the first successful treatment of COVID-19-associated ARDS using ruxolitinib. COVID-19 is characterized by an exuberant inflammatory response [7] that can result in massive lung injury (ARDS), multiorgan failure and death. COVID-19-associated ARDS requiring invasive ventilation is characterized by low survival rates—especially in older patients [7].

The decision to treat our patient with ruxolitinib to inhibit JAKs was based on the current knowledge of COVID-19 pathophysiology, which is thought to be mediated by an overwhelming inflammatory cytokine response and thus is very likely to involve JAK-signaling. We reasoned that by blocking JAK1/2-kinases the devastating consequences especially of inflammatory lung tissue damage could be diminished. The use of ruxolitinib as an immunosuppressive agent is not without precedence: in steroid–refractory graft versus host disease (GvHD)—an aggressive form of organ-damaging, cytokine-mediated hyperinflammation after allogeneic hematopoietic stem cell transplantation, ruxolitinib resulted in impressive clinical improvements [8].

In our patient, ruxolitinib not only potently reduced ARDS-associated inflammatory blood cytokine levels such as IL-6 and the acute phase protein ferritin, but was also associated with a rapid respiratory and cardiac improvement and clinical stabilization. This course was remarkable when compared to other patients [6]. Based on the close temporal association between ruxolitinib start and clinical response, it is tempting to speculate that JAK1/2-inhibition effectively contributed to the favorable clinical course. Importantly, the virus load as determined by PCR did not increase in our patient during ruxolitinib treatment.

Artificial intelligence and pre-clinical studies have recently suggested that baricitinib, a numb associated kinase (NAK) and JAK1/2-inhibitor, inhibits clathrin-mediated endocytosis and thereby antagonizes SARS-CoV-2 infection of cells [9]. Thus, besides ruxolitinib, baricitinib, which is approved in the treatment of rheumatoid arthritis, is another promising candidate with significant potential in the treatment of COVID-19 disease. However, since baricitinib may not penetrate into lung tissue [10], we hypothesized that it may be less effective in COVID-19-associated ARDS.

The finding in this patient may have important implications for the ongoing search for optimal therapy for patients suffering from severe COVID-19. Clinical trials are under way to study both ruxolitinib and baricitinib in a prospective manner (e.g. NCT04359290).
